# Silent Invader: A Rare Case of Enterobacter aerogenes Empyema in a Hospice Patient With Complex Comorbidities

**DOI:** 10.7759/cureus.73397

**Published:** 2024-11-10

**Authors:** Hansani Angammana, Kafayat Omadevuae, Victoria Bengualid, Rawand Khader

**Affiliations:** 1 Internal Medicine, Saint Barnabas Hospital Health System, New York, USA; 2 Infectious Diseases, Saint Barnabas Hospital Health System, New York, USA; 3 Geriatrics, Saint Barnabas Hospital Health System, New York, USA

**Keywords:** antibiotic, aspiration, empyema, enterobacter, lung abscess

## Abstract

*Enterobacter aerogenes* (recently renamed *Klebsiella aerogenes*) is an uncommon pathogen in pleural infections and empyema, typically associated with nosocomial urinary and gastrointestinal infections. This case report describes a 69-year-old male patient with chronic kidney disease, diabetes mellitus, and other comorbidities, who developed empyema despite broad-spectrum antibiotics. Pleural fluid cultures revealed *E. aerogenes*, known for its ability to develop resistance through beta-lactamase production and efflux pumps, which complicates treatment. Despite initial improvement with cefepime and metronidazole, the patient's respiratory status deteriorated, and due to his do not resuscitate/do not intubate (DNR/DNI) status and extensive comorbidities, no further aggressive interventions were pursued, leading to his passing. This case highlights the diagnostic and therapeutic challenges posed by *E. aerogenes* in pleural infections, emphasizing its rarity in pulmonary involvement and its potential for antibiotic resistance. It also underscores the importance of considering atypical pathogens in complex infections and the need for multidisciplinary management while balancing aggressive treatments with patient-centered care, particularly in end-of-life scenarios.

## Introduction

Empyema is a serious complication of pleural infections, commonly caused by bacterial pathogens such as *Streptococcus pneumoniae* and *Staphylococcus aureus*. However, *Enterobacter aerogenes*, recently reclassified as *Klebsiella aerogenes* [1], is an exceedingly rare pathogen in pulmonary infections and empyema. Most reported cases of *E. aerogenes* infections are associated with urinary tract infections, gastrointestinal infections, or bacteremia in immunocompromised patients. The presence of this organism in pleural infections is uncommon and presents unique diagnostic and therapeutic challenges [2], particularly in patients with multiple comorbidities.

In this case, we present a patient with advanced comorbidities, including chronic kidney disease (CKD), diabetes mellitus, cerebrovascular accident (CVA), and peripheral artery disease, who developed empyema due to *E. aerogenes*. Despite the initial management with broad-spectrum antibiotics and drainage interventions, the patient's condition deteriorated, highlighting the challenges in managing such atypical infections in complex patients [3-6]. This case emphasizes the need for early identification and aggressive management of rare pathogens and the importance of multidisciplinary care. Furthermore, it raises important questions about the role of *E. aerogenes* as an emerging pathogen in respiratory infections and the potential resistance mechanisms that complicate its treatment.

## Case presentation

**Figure 1:**
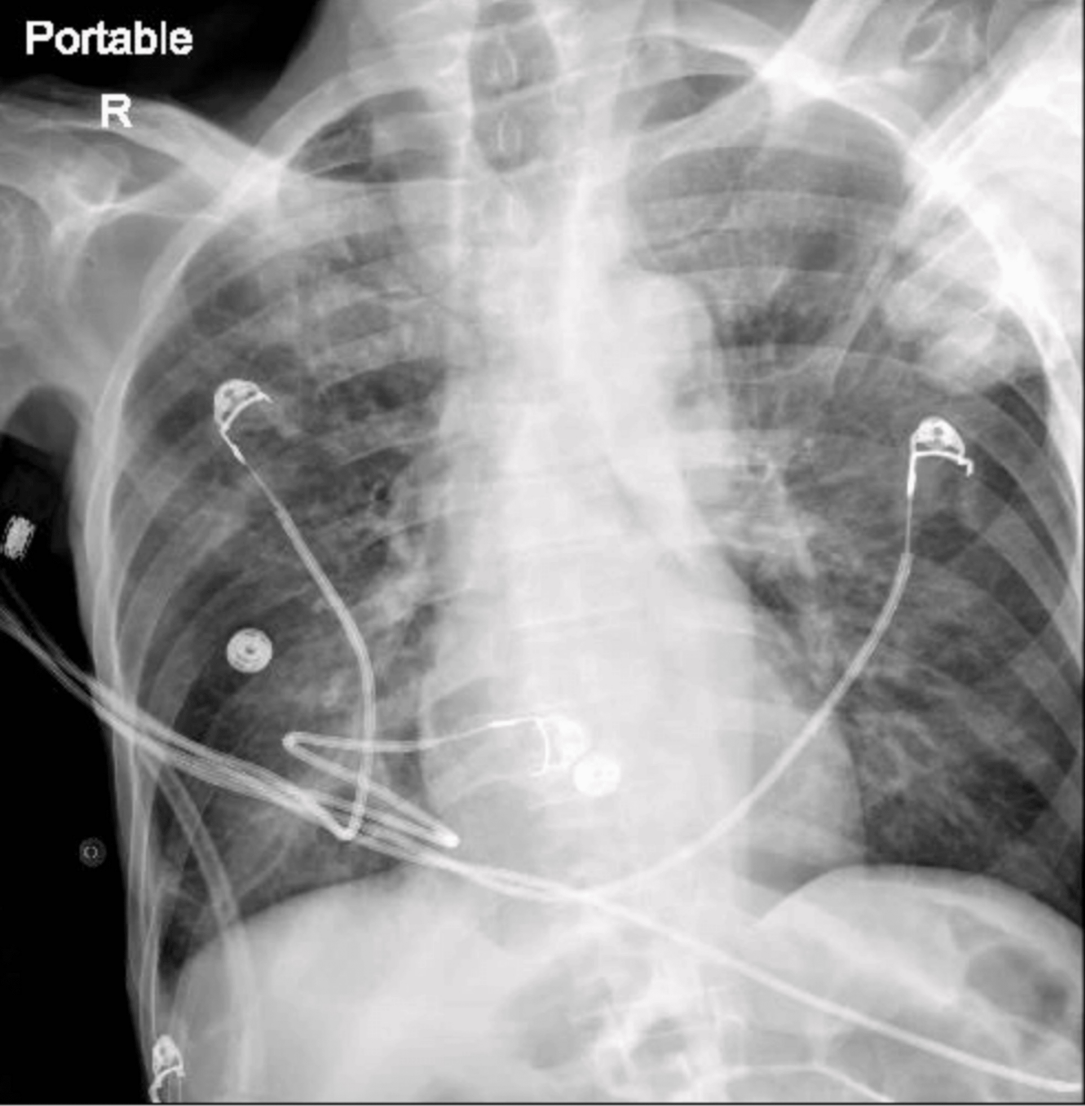
Chest XR on admission showing coarse bronchovascular markings. XR: X-ray

A 69-year-old male patient previously in hospice care-with a history of CKD, diabetes mellitus, hypertension, prior CVA, and peripheral artery disease status post-bilateral below-knee amputation-was admitted following acute mental status changes and hypoxia. His baseline was advanced dementia, and he was nonverbal and bed-bound. Upon presentation, he was hypoxic with oxygen (O_2_) saturation in the high 80s, which improved with 3 L oxygen. The laboratory findings (see Table 1) revealed leukocytosis, anemia, elevated lactic acid, and renal dysfunction. The chest X-ray (CXR) showed coarsened bronchovascular markings (see Figure 1), and broad-spectrum antibiotics including vancomycin, ceftriaxone, and azithromycin were initiated.

**Table 1:** Laboratory findings on admission.

Test	Observed value	Reference range
White blood cells	11.6 x 10^3^/µL	4.0-11.0 x 10^3^/µL
Hemoglobin	9.3 g/dL	12.1-15.1 g/dL
Lactic acid	2.3 mmol/L	0.5-2.0 mmol/L
Creatinine	2.3 mg/dL	0.7-1.3 mg/dL
Blood urea nitrogen	89 mmol/L	2.1-8.5 mmol/L

**Figure 2:**
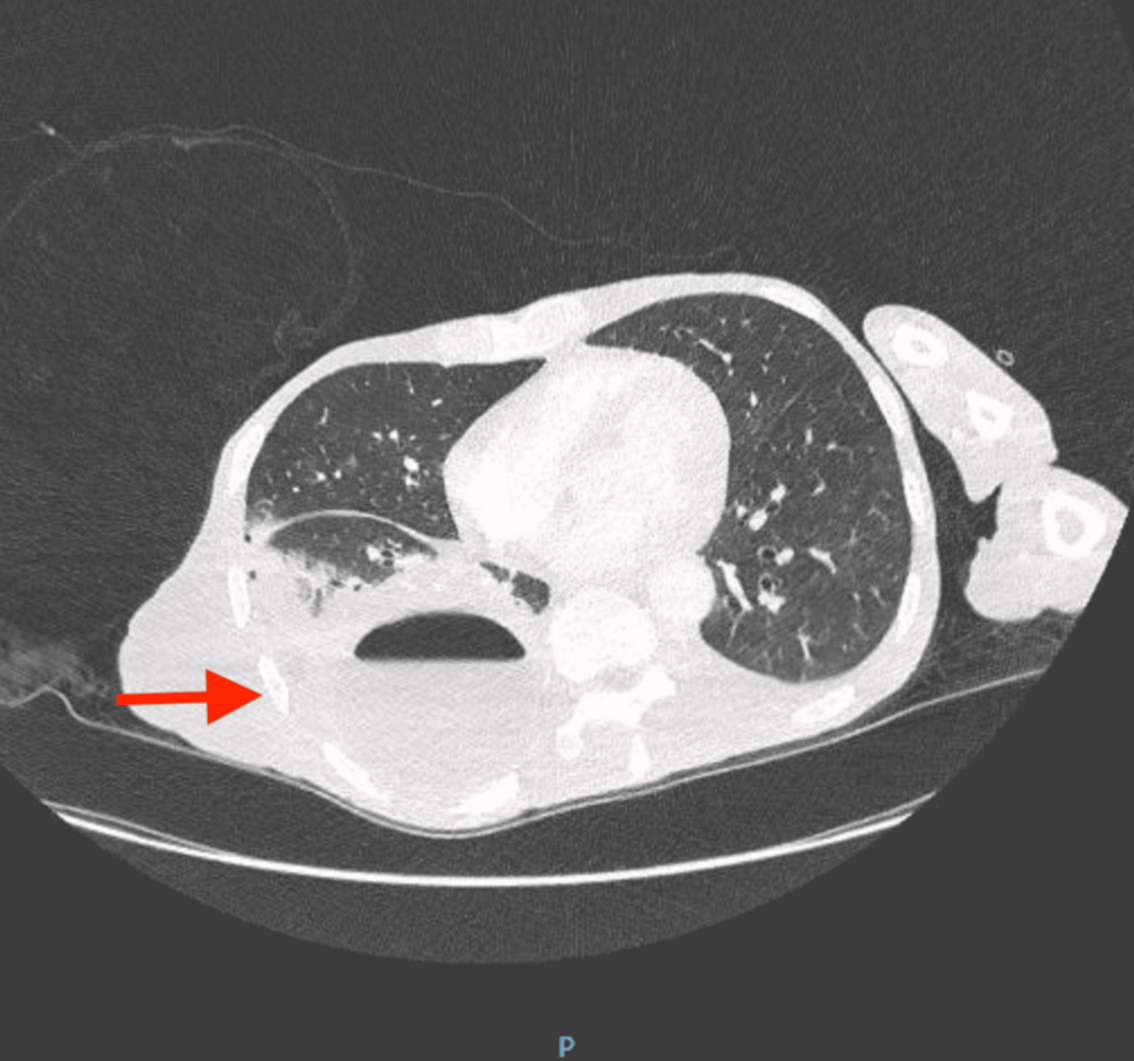
CT chest showing multiple loculated fluid collections in the right lower lobe.

Despite initiating antibiotic therapy, the patient's clinical condition deteriorated, which was evidenced by (1) an increasing white blood cell (WBC) count (reaching 22.6 × 10^3^/μL) and (2) concerning imaging findings in computed tomography (CT) of the chest that revealed multiple loculated fluid collections in the right lower lobe (see Figure 2), consistent with empyema. This led to the escalation of medical management to a consult of infectious disease, who recommended a change from ceftriaxone to cefepime and an addition of Flagyl on Day 6.

At this point, the pulmonary team was involved in the care, and a referral to cardiothoracic surgery for possible video-assisted thoracoscopic surgery (VATS) or decortication was recommended. This was deemed unfeasible given the patient’s numerous comorbidities and inability to tolerate surgery. Therefore, a right-sided chest tube was placed by interventional radiology, and pleural fluid was collected, cultures of which identified *E. aerogenes*.

Despite ongoing broad-spectrum antibiotics (including cefepime and Flagyl) and supportive care, the patient’s condition remained tenuous. The repeat CXRs showed an interval reduction in the size of empyema, particularly with decreased gas within the fluid collection. His WBC count also improved, decreasing to 8.8 × 10^3^/μL. However, on Day 20, the patient's respiratory status worsened, requiring bilevel-positive airway pressure (BiPAP). Due to his do not resuscitate/do not intubate (DNR/DNI) status, neither intubation nor aggressive interventions were pursued. The patient passed away the same day.

## Discussion

Empyema due to *E. aerogenes* (recently renamed *K. aerogenes* [1]) is an exceedingly rare clinical finding. While *Enterobacter* species are more commonly associated with urinary and gastrointestinal infections, their role in pulmonary infections, particularly empyema, remains uncommon. This case underscores the diagnostic difficulties posed by atypical pathogens in patients with advanced comorbidities, such as CKD and diabetes. The loculated nature of the empyema further complicated the management, as drainage interventions such as thoracentesis were deemed likely unsuccessful [2].

One of the notable pathogenic features of *E. aerogenes* is their ability to develop resistance to a wide range of antibiotics, particularly through the production of beta-lactamases [3] (which hydrolyze beta-lactam antibiotics), and efflux pumps that contribute to multidrug resistance. These resistance mechanisms often complicate the management of infections caused by *E. aerogenes*, necessitating broad-spectrum antibiotics [6] and combination therapies in critically ill patients.

The development of *E. aerogenes* empyema in this case was likely a result of chronic aspiration [4], a common issue in patients with neurocognitive impairments or those who are nonverbal. Aspiration allows bacteria from the oropharynx to enter the respiratory tract, potentially leading to infection. Given the patient’s history of CVA, his compromised swallowing function made him susceptible to micro-aspiration over time.

The decision to continue conservative management, including antibiotics and chest tube drainage, was guided by the patient’s stable clinical status and the low likelihood of procedural success. The involvement of a multidisciplinary team-comprising pulmonology, thoracic surgery, and infectious disease-highlighted the complexity of managing such cases [5].

## Conclusions

This case highlights the rare occurrence of *E. aerogenes* as a causative agent of loculated empyema, particularly in a patient with multiple comorbidities. The clinical presentation, characterized by progressive respiratory distress and imaging findings of loculated fluid collections, underscores the complexity of managing empyema in patients with significant underlying health issues. Despite the initiation of broad-spectrum antibiotic therapy, the patient's condition necessitated a multidisciplinary approach involving pulmonology and thoracic surgery, emphasizing the need for timely surgical intervention in cases where traditional drainage methods may be ineffective. This case serves as a reminder to clinicians to consider atypical pathogens in the differential diagnosis of empyema, particularly in patients with a history of chronic illness. Future studies should further explore the epidemiology and management strategies for infections caused by *E. aerogenes*, aiming to improve outcomes for patients with similar clinical presentations.
